# Genital Herpes: Insights into Sexually Transmitted Infectious Disease

**DOI:** 10.15698/mic2016.09.528

**Published:** 2016-06-27

**Authors:** Dinesh Jaishankar, Deepak Shukla

**Affiliations:** 1,2 Departments of Bioengineering and Ophthalmology and Visual Sciences, University of Illinois at Chicago, IL 60612.; 3 Department of Microbiology and Immunology, University of Illinois at Chicago, IL 60612.; 4 Department of Pathology, University of Illinois at Chicago, IL 60612.

**Keywords:** herpes simplex virus, virus entry, viral glycoproteins, viral latency, antivirals

## Abstract

Etiology, transmission and protection: Herpes simplex virus-2 (HSV-2) is a leading cause of sexually transmitted infections with recurring manifestations throughout the lifetime of infected hosts. Currently no effective vaccines or prophylactics exist that provide complete protection or immunity from the virus, which is endemic throughout the world. Pathology/Symptomatology: Primary and recurrent infections result in lesions and inflammation around the genital area and the latter accounts for majority of genital herpes instances. Immunocompromised patients including neonates are susceptible to additional systemic infections including debilitating consequences of nervous system inflammation. Epidemiology, incidence and prevalence: More than 500 million people are infected worldwide and most reported cases involve the age groups between 16-40 years, which coincides with an increase in sexual activity among this age group. While these numbers are an estimate, the actual numbers may be underestimated as many people are asymptomatic or do not report the symptoms. Treatment and curability: Currently prescribed medications, mostly nucleoside analogs, only reduce the symptoms caused by an active infection, but do not eliminate the virus or reduce latency. Therefore, no cure exists against genital herpes and infected patients suffer from periodic recurrences of disease symptoms for their entire lives. Molecular mechanisms of infection: The last few decades have generated many new advances in our understanding of the mechanisms that drive HSV infection. The viral entry receptors such as nectin-1 and HVEM have been identified, cytoskeletal signaling and membrane structures such as filopodia have been directly implicated in viral entry, host motor proteins and their viral ligands have been shown to facilitate capsid transport and many host and HSV proteins have been identified that help with viral replication and pathogenesis. New understanding has emerged on the role of autophagy and other innate immune mechanisms that are subverted to enhance HSV pathogenesis. This review summarizes our current understanding of HSV-2 and associated diseases and available or upcoming new treatments.

## INTRODUCTION

Genital herpes is one of the most common, persistent and highly infectious sexually transmitted viral infections mostly caused by herpes simplex virus-2 (HSV-2) and in many emerging first time cases, by HSV-1 1. Primary and recurrent genital herpes infections most commonly result in lesions and inflammation around the genital area. In women, the sites of infection are mainly the vulva and the vagina, with some cases involving the regions of cervix and perianal. In heterosexual men infection is typically on the glans or the shaft of the penis, whereas anal infection is also reported with homosexual men. More than 500 million people are infected worldwide and most cases reported are among the age groups between 16-40 years that coincides with increased sexual activity among this age group 2. While these numbers are an estimate, the actual numbers may be underestimated as many people are either asymptomatic or are unaware of the infection 3. This review provides an insight into the epidemiology, pathology, our current understanding of the molecular mechanisms of infection and the currently available and upcoming treatments for genital herpes.

## EPIDEMIOLOGY AND PREVALENCE

Herpesviruses are among the most ubiquitous of human infections. After infection with HSV, it is thought that the virus and the immune response to the virus persist through the life of the host. HSV infections are measured by testing various populations for the presence of antibodies specific to the virus. An estimated 90% of all people worldwide have one or both viruses 4,5. HSV-1 is the more prevalent virus with 65% of persons in the United States having antibodies to HSV-1 6, while HSV-2 infections are markedly less frequent, with 15%-80% of people in various populations infected 7. HSV-1 and HSV-2 infection rates widely vary between countries. The increase in genital HSV-1 is mainly attributed to an increase in oral sex among youngsters and adults which is viewed safer than intercourse 8. Due to this, in the USA, Canada, and other European countries, at least half of the first episodes for genital herpes have been caused by HSV-1 in the past decade 9,10,11,12. In a study performed by the CDC it is estimated that about one in six Americans aged 14 to 49 are infected with HSV-2 and the prevalence in women was 20.9%, twice as high as among men 13. While a surge of HSV-2 seroprevalence from 16.4% to 21.8% was observed from 1976 to 1994 14, this trend has reversed, dropping to 17.2% in 2004 15. In Africa and other developing countries, there is a high burden of HSV-2 infections with >50% prevalence in the population 16. Around 82% of women and 53% of men in the Sub-Saharan Africa are seropositive for HSV-2 17. HSV-2 infection rates also depend on the rates of sexual activity and are more prevalent in heavily exposed populations, such as commercial sex workers, who are nearly 100% positive, suggesting an urgent need for education and new measures for prevention 18.

## MOLECULAR MECHANISMS OF INFECTION

**Figure 1:**
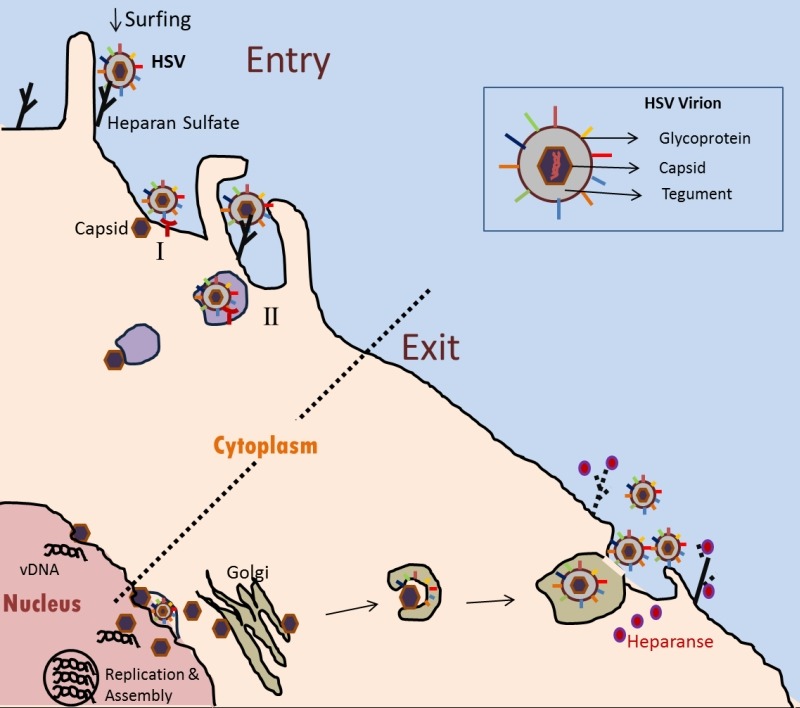
FIGURE 1: Schematic of HSV-1/HSV-2 lytic infection. The HSV-1/HSV-2 virion recognizes and attaches to the heparan sulfate proteoglycan via glycoproteins on the viral envelope. By a process called ‘surfing’, the virus particles can travel along filopodia-like membrane extensions to reach the surface of the cell. On the surface of the cell, viral capsid penetration can occur by fusion of envelop with the plasma membrane (I), or alternatively by endocytosis of enveloped virions with eventual fusion of the envelope with a vesicular membrane (II). In either case, gD on the virus envelope is required via its interaction with one of the receptors (shown in red): herpesvirus entry mediator (HVEM) or nectin-1 and-2. In the cytoplasm, the capsid (brown) travels to the nucleus where the viral DNA is released. Multiple rounds of replication result in multiple copies of viral DNA and other components that get packaged and assembled in the nucleus. During egress, the newly assembled capsid gets its primary envelope at the peri-nuclear membrane, which is lost during egress from the outer nuclear membrane. Naked capsid travels through the cytoplasm where it receives the tegument and the viral envelope (presumably from the Golgi or the ER). Heparanase (denoted as pink spots) is an enzyme that was recently described in aiding viral egress. The enzyme cleaves of cell surface heparan sulfate (dotted black) which clears the path for the virus to exit the cell.

HSV are linear, double stranded DNA viruses capable of establishing latency in humans. They belong to the family of Herpesviridae and more specifically to the sub-family of Alphaherpesvirinae. There are two sub-types: HSV-1 and HSV-2 that are closely related but differ slightly in tissue tropism and antigenic properties. The viral DNA is present in the core that is enclosed in a protein shell called the capsid (Fig. 1). The icosahedral shaped capsid is ~125 nm in diameter, which is connected to and surrounded by a glycoprotein expressing lipid bilayer membrane envelope via a protein coat called the tegument. The viral envelope contains at least 12 glycoproteins many of which play major roles in the entry and egress of the virus. A list of HSV glycoproteins along with their reported functions is provided in Table 1.

**Table 1:** List of HSV glycoproteins and their reported functions.

**Glycoprotein**	**Function**	**References**
gB	Fusogenic protein: class III	23
gC	Attachment and C3b receptor	20,24
gD	Virus entry and fusion	19,21
gE	Virus spread and Fc receptor	22,25
gH	Virus entry and fusion	19,21
gI	Virus spread and Fc receptor	22,26,27
gK	Virus spread and egress	28,29
gL	Viral entry and fusion	19,24
gM	Virus assembly and fusion	30,31,32

The lifecycle of HSV has been mostly studied and characterized using HSV-1 infections. However, HSV-2 infections are considered similar to HSV-1 infections. Different stages in the HSV lifecycle can be broadly classified into:

**i. Attachment:** Initiation of infection begins with the attachment of viral glycoproteins to the cell surface. Heparan sulfate proteoglycans (HSPGs) on the cells serve as attachment sites for HSV 19. Glycoproteins B and C (gB and gC) on the HSV envelop bind to the HSPGs and are essential to initiate attachment. A study by Herold *et al.*, using a gB and gC null virus showed reduction in the overall virus attachment to the cells as well as reduction in virus infectivity 20. Moreover, it has been shown that in the absence of gC gB can take over and help in attachment to cells, indicating a gC-independent mode of viral attachment 33. HSV was shown to bind to HS (heparan sulfate) on the filopodia, which are plasma membrane protrusions, and use filopodial interaction to migrate towards the cell body to initiate entry. This process was termed "viral surfing" 34. In this study, viral particles were shown to surf along the filopodia and the formation of filopodial structures increased upon HSV infection, possibly due to activation of Rho GTPase signaling during virus attachment to cells. Fluorescence imaging revealed that HSPG expression is higher along the filopodial structures. This mode of attachment has also been reported for vaccinia virus, human papilloma virus type 16, hepatitis C virus, and human immunodeficiency virus (HIV) 35.

**ii. Entry:** After the initial attachment to the cell surface, virus entry is the next step in the lifecycle. Various modes of viral entry have been established. The virus is taken into the cells by either direct fusion with the plasma membrane, which is independent of pH change, or through endocytosis mediated by specific cellular receptors. The glycoprotein D (gD) on HSV plays an important role in both of the aforementioned uptake processes and glycoproteins H and L (gH and gL) act in concert to complete the fusion machinery. To date the following receptors have been identified for gD: herpes virus entry mediator (HVEM), nectin-1 and -2 and 3-O sulfated heparan sulfate (3-OS HS) 21. HVEM was the first identified HSV receptor that belongs to the tumor necrosis factor (TNF) superfamily. The next set of receptors identified is represented by nectin-1 and -2. They belong to the immunoglobulin superfamily. The last receptor is a rare modification of the large sugar molecule HS mediated by the 3-O-sulfotransferase 3 (3-OST-3). 3-OST-3 belongs to the family of 3-O sulfotransferases (3-OSTs) that place sulfate groups at the 3-OH position on the glucosamine in HS. This specific and rare modification of HS dictates the biological activity of HS and occurs during the last step of HS biosynthesis. As an example, modification of HS by 3-OST-1 serves as a binding site for antithrombin, a major player in anticoagulation 36. 3-OST-3 modified HS serves as an entry receptor for HSV and addition of soluble form of 3-OS HS in HSV resistant cell lines showed increased viral entry 38,39. Interestingly, 3-OST-3 generated receptor fails to mediate HSV-2 entry but may probably help in the attachment of HSV-2 19,38.

Viral entry can occur in the presence of any one of the aforementioned receptors and absence of all three receptors abolishes viral entry. Even though gD is needed for receptor-mediated endocytosis and also for the direct fusion of viral envelop to the plasma membrane, there seems to be no clear consensus on how and which mode of entry the viruses use in human hosts or animal models. While entry into some cultured cells like CHO, HeLa and HCEs are reported to be through receptor mediated endocytosis, entry into Vero and neuronal cell lines are through direct fusion with the plasma membrane 39,40. In addition to gD playing a vital role in viral entry, accumulating evidence also suggests the important role of gB in HSV entry as a gB null virus was unable to enter and cause infection in target cells 41. Paired immunoglobulin-like type 2 receptor α (PILRα) has been shown to associate with gB to function as a co-receptor in aiding HSV-1 entry. Mutations on the sites where gB attaches to PILRα not only reduced viral entry but also reduced viral replication and neuroinvasiveness 42,43,44. Furthermore, another protein that belongs to the sialic acid-binding Ig-like lectin family which shares a similar homology to PILRα called the myelin-associated glycoprotein (MAG) acts as a co-receptor for HSV-1 entry when expressed exogenously 45. Another co-receptor called non-muscle myosin IIA (NMIIA) was also identified to bind gB on the cell surface and aide in the viral entry 46. As an actin binding motor protein, NM-IIA plays a critical role in cell adhesion and migration. The glycoproteins gH and gL together with gB and gD form the fusion complex 47,48. gH exists as a hetero-oligomeric complex with gL. This complex is essential for the processing and cell surface expression of gH 49,50 and is conserved in many of the herpesviruses 51. Apart from playing a role in the fusion machinery, the gH/gL complex plays a role in virus entry by interacting with various cell surface proteins 52, integrins being the most common. Interaction of gH with integrin αvβ3 facilitates HSV-2 viral entry and calcium signaling in human genital tract epithelial cells 53. Another study shows that αvβ6 and αvβ8 serve as interchangeable receptors for gH/gL that promote endocytosis and activation of membrane fusion 54. A recent study by the same group also found that conformational changes in the above mentioned integrin receptors are essential to promote the dissociation of gL from the gH/gL complex, a proposed new mechanism in HSV viral entry 55.

Other alternative modes of viral entry have also been identified. A phagocytosis-like uptake of the virus particles was reported to be observed once the virus particles have attached to the filopodia; it is believed to exhibit mixed traits of endocytosis and phagocytosis 56. Cytoskeleton rearrangement and their associated cellular signaling pathways have also been implicated in facilitating HSV entry into cells 57. Rho-GTPase signaling pathway involving Rho-A and cdc42, key modulators in the formation of filopodia, were shown to be activated and aide in the phagocytic-like uptake of the virus 56. Another signaling pathway called phosphoinositide 3 kinase (PI3K) pathway, which is involved in the downstream of the filopodial formation, was also found to affect multiple steps in the HSV entry 58. This same pathway is also implicated to control the activity of cofilin, a family of actin-binding proteins, in facilitating entry of virus into neuronal cells 59. The activation of Akt signaling in triggering calcium release which aids in HSV viral entry has also been shown 60.

**iii. Capsid Transport and Replication:** Upon successful entry into cells, the viral capsid and tegument proteins are released into the cytoplasm. The virion host shutoff protein (vhs) is a viral tegument protein that is released into the cytoplasm after entry and degrades host mRNAs that regulate stress response. The capsid then translocates to the nucleus along microtubules via the dynein and dynactin motor proteins and releases the viral DNA into the nucleoplasm 61,62,63. A recent study reported the role of heat-shock protein 90 (Hsp90) to be involved with HSV capsid transport to the nucleus via interaction with acetylated α-tubulin 64. The uncoating of viral DNA occurs at the nuclear pore.

**iv. Replication and Assembly:** Once inside the nucleus, several viral genes are expressed in an ordered fashion. The proteins of the α genes or intermediate early (IE) genes are the first to be transcribed. The products of these genes are termed as infected cell protein (ICP) and there are five ICPs: 0, 4, 22, 27 and 47. The virus encodes a tegument protein: VP16 that aids in the transcription of the α genes. The expression of ICP4 is then thought to drive the expression of the β genes or the early genes. The β genes encode for various proteins that promote viral DNA replication, including the enzyme thymidine kinase (TK). The virus utilizes TK for replication leading to the expression of the γ or late genes. The proteins of the γ genes encode for several components of the viral structure including capsid and envelop proteins. Various viral components are formed which then assemble and the viral DNA is repackaged into a new capsid. Fully assembled capsid exits from the nucleus by acquiring a glycoprotein-containing envelop at the inner nuclear membrane and losing it at the outer membrane when the naked capsid is released in the cytoplasm for re-envelopment using a Golgi-derived membrane (Fig. 1).

**v. Autophagy Modulation during Active Replication:** The role of autophagy, a cellular process involved in maintaining the metabolic and homeostatic activity, in HSV replication has been widely studied. The ICP34.5 protein, a neurovirulence factor, regulates the replication of HSV by controlling the autophagic pathway via inhibition of either PKR/eIF2a signaling pathway 65,66 or beclin-1, a protein involved in the formation of autophagosomes 67. A recent study showed that a basal level of autophagy is needed for efficient replication of virus and disrupting the basal level would lead to reduced viral titers 68. Another recent study showed the role of a host cytoplasmic protein called axin in controlling autophagy and HSV replication 69. The results from this study indicate that axin expression reduces the levels of cellular autophagy induced by HSV, resulting in enhanced HSV replication.

**Figure 2:**
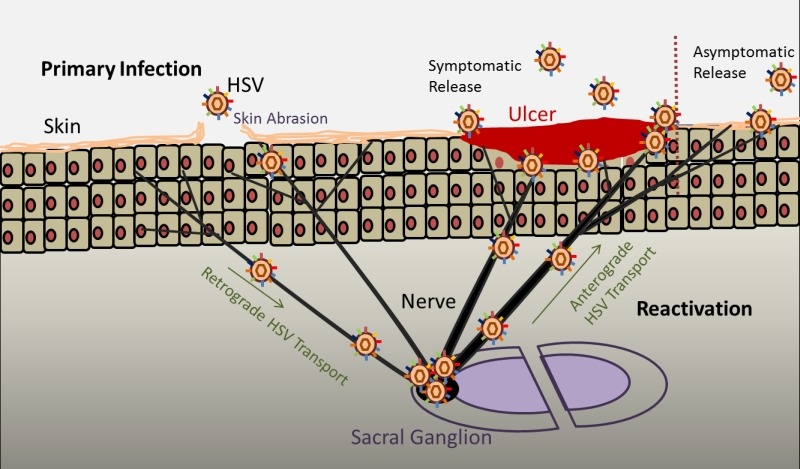
FIGURE 2: Schematic of Primary Infection and Reactivation. Primary infection occurs when a host is exposed to the virus for the first time. When a person is exposed to HSV, the virus infects the epithelial cells. Depending on the immune system of the host, lytic infection leads to virus shedding that can cause symptoms such as ulcers or remain asymptomatic. After lytic infection, the virions reach the nerve endings and through a retrograde transport, reach the sacral ganglion where it establishes latency till the life of the host. Recurrent infections occur when the virus gets reactivated due to stress, environmental conditions and other unknown factors. Reactivation causes the virus from the sacral ganglion to travel to the site of primary infection or high nerve endings via an anterograde fashion where virus shedding can cause symptoms or remain asymptomatic depending on the host immune system.

**vi. Latency and Reactivation:** One of the key traits of this family of viruses is to go latent for the life of the host after primary infection. How and why the virus goes latent is only partially understood and is one of the hot topics in herpes research. After a lytic infection the virus has the ability to evade and mask itself from the host defense. Latency is established when the virus migrates to the sensory ganglia via a retrograde fashion and invades the nucleus of the neurons (Fig. 2). In the nucleus the HSV genome is maintained in a circular form and remains in a silent state. During this state, a region of the genome that encodes for the latency associated transcripts (LATs) remains active 70. Kramer *et al*. also showed the presence of HSV transcripts using RT-PCR analyses in latently infected mouse ganglia 71,72. The exact role and function of the LATs also remains to be completely understood. However, research over the last decade has revealed the common functions of LAT: they help in reducing the expression of the viral genome thereby maintaining them in a latent state protected from the immune system 73 and they protect infected neurons from apoptosis, thus increasing the amount of latent transcripts that would eventually increase the viral load upon reactivation 74,75. In addition, the host immune system has also been implicated to play a vital role in viral latency. Studies in the mouse models of latent HSV infection revealed the presence of infiltrating immune cells and cytokines in latently infected ganglia 76,77,78 while some suggest that the presence of low viral transcript levels could lead to a local milieu of immune effectors that could repress HSV gene expression 79,80. Some evidence also suggests the role of neuronal functio in maintaining latency 81,82,83. Furthermore, during latent infection, the ability of some parts of the HSV genome to remain transcriptionally active and inactive suggested the presence of epigenetic control. Two studies that used computational analysis and latently infected mice revealed that DNA methylation, a most common epigenetic mechanism, did not regulate HSV latent gene expression 84,85, leading to the investigation of other epigenetic mechanisms.

The role of chromatin and HSV latency has gained increasing popularity as the HSV DNA is devoid of histones 86 but upon infection gets assembled into the nucleosome 87 and associates with histones 88. However whether heterochromatin or euchromatin play a role in HSV latency was not known. Only recently, using various molecular techniques, the presence of heterochromatin or euchromatin in HSV-infected cells has been studied to provide a basis for a chromatin-based epigenetic mechanism of HSV gene regulation in different cell types 89. In a study by Kubat *et al*., their findings showed that active chromatin was associated with LAT gene as increasing levels of acetylated H3 histone were found to be associated with the LAT promoter and enhancer compared with the ICP0 gene 90. In another study by Wang *et al*., it was shown that as latent infection is established the HSV lytic genes are progressively associated with chromatin that contains dimethylation of H3K9me2, which is an indicator of heterochromatin 91. Thus there is a general notion that during latent infection the LAT gene is associated with euchromatin whereas the lytic genes are associated with heterochromatin. The study by Amelio *et al*. gave insights into how and why different chromatin are maintained and regulated separately on the latent viral genome 92. Their study identified candidate insulator elements, DNA sequences that bind protein factors that maintain chromatin boundaries. These contain CCCTC sites that are bound by the CCCTC-binding factor (CTCF) upstream of the LAT promoter boundary and in the LAT intron. They proposed that insulators keep the LAT euchromatin activity within a boundary and heterochromatin outside of the same boundary.

Reactivation of the latent virus occurs when an external stimuli or ‘stress’ is applied to the neuron. Various factors such as environmental conditions, fever, exposure to sunlight and other unknown conditions have been attributed to cause reactivation but their exact targets at the molecular level remain unknown. When the virus reactivates it travels from the sensory ganglia via anti-retrograde fashion to the primary infection site or sites of high neuron innervations where active virus replication and shedding occur and symptoms like pain, inflammation and lesions develop. In an effort to understand the exact role of LATs in the reactivation of HSV, LAT encoded micro RNAs (miRNA) were discovered. miRNAs are a family of non-coding RNA that is approximately 22 nucleotides in length. They usually function at a post-transcription level by inhibiting protein synthesis via mRNA degradation. HSV miRNAs have been shown to be expressed during productive infection, which helps degrade host immune responses as well as during latency, which helps in establishing latency or helps in reactivation 93.

**vii. Egress:** Upon formation of capsid and packaging of the virus DNA, the virions eventually have to egress or leave the nucleus and the cell to get into the extracellular environment. While the process of HSV egress still requires some clarity due to varying experimental models and complexity in studying the virus-nuclear interactions, the following is the accepted model for viral egress. Budding is the initial step in the nuclear egress of HSV. In this process the capsid acquires the envelope from the inner nuclear membrane and two viral proteins: UL31 and UL34 are reported to be necessary for the budding process 94. Once the virus reaches the perinuclear region, it is thought to lose the primary envelope or undergoes de-envelopment and evidence suggests that the final assembly of tegument, envelope and the glycoproteins occur within the cytoplasmic compartments (presumably in the Golgi or Endoplasmic Reticulum, ER). During productive infection either in primary infection or after reactivation, for efficient transmission and infection, the virus needs to spread to neighboring cells. The release of the virus from infected cells requires both host factors and viral components. Among the viral components, glycoproteins E and I (gE and gI) are needed for efficient spread of viruses in certain polarized and non-polarized epithelial cells and neuronal cells 95,22. Among the host factors, a HS degrading enzyme: heparanase (HPSE) has been recently shown to aide in viral egress 96. The study shows how the levels of HPSE increase over time with HSV infection as active form of HPSE is translocated to the plasma membrane of infected cells to remove HS for smoother release of newly generated virions. The role of myosin motor proteins such as NMIIA and myoVa have also been implicated in HSV egress 97,98.

## SYMPTOMS AND PATHOLOGY

Genital herpes is predominantly transmitted through sexual contact. Viral transmission by oro-genital contact is mostly HSV-1 and therefore the number of genital HSV-1 cases is on the rise 99,100,101. Virus shedding is more predominant in sites like mouth and mucosal surfaces such as the vagina. Contact with any one of these increases the risk of being infected with HSV.

An episode or outbreak is termed as the phase in which individuals experience symptoms and the severity of these episodes depends on previous immunity to HSV. Notably, almost 25% of people presenting with a first clinical episode of genital herpes have serological evidence of past HSV-2 infection at the time of presentation, suggesting initial infection was asymptomatic 102. In many other instances of primary infections where the patient encounters HSV for the first time the first episode may occur anywhere between 2 days to 2 weeks after primary infection. Primary infections are clinically most severe and most likely symptomatic 103. Symptoms like fever, itching and muscle pains usually in the lower part of the body are most common in primary infection; 40% of men and 70% of women also report fever, headache, malaise, and myalgias 104. Papule formulation followed by a wide distribution of blisters or lesions appear around the genital areas that eventually break to form ulcers (Fig. 2). Over a period of time the ulcers crust and heal. In women common sites for lesion are the cervix, vagina, labia majora and minora and perianal region through infected vaginal fluid and in men it is mostly on the shaft or the glans of the penis. Anal lesions are also reported in homosexual men. Primary infections either by HSV-1 or by HSV-2 cannot be differentiated just by clinical symptoms; additional laboratory testing is needed to differentiate between the two viruses.

At the tissue and molecular level, HSV-2 infects the epithelial cells on the genital mucosa leading to an increase in inflammatory response and cell death at the site of infection. Multinucleated cells and syncytia formation are the most common observation in cells infected with HSV. The recruitment of macrophages, natural killer cells, B-cell and T-cell mediated immunity 105,106 and the release of cytokines has been reported to play a role in innate and adaptive immunity to HSV infections. This contributes to a chronic inflammatory state in genital skin and mucosa. Histopathologic studies of foreskin in HIV-seronegative men after adult circumcision have shown a higher concentration of CD4^+^ and CD8^+^ T-cells in HSV-2-seropositive compared with HSV-2-seronegative men 107. During the course of primary infection, the virus spreads via a retrograde fashion along the microtubules lining the axons to the dorsal root ganglia (DRG) where the neuronal cells act as reservoirs for the virus to remain latent 108. Upon reactivation due to factors such as stress and other unknown conditions, the virus spreads from the DRG to the epithelial cells via an anterograde fashion where a lytic replication of the virus follows, resulting in virus shedding. This is the cause of recurrent infections and these infections are usually asymptomatic or may be associated with a classic genital ulcer. While the innate immune system, specifically the CD8^+^ T-cells and the plasmacytoid dendritic cells, are attributed in controlling latency and reactivation of the virus 80,109,110, recent reports suggest otherwise. Studies have shown that CD8α dendritic cells help drive the establishment of HSV-1 latency 111,112. At a clinical and subclinical level, the severity of viral reactivation varies widely from person to person and depends on cell mediated immunity that is considered important for control of viral replication 113,114.

## DIAGNOSIS

Diagnosis of genital herpes based purely on clinical presentation is often not accurate and could be misleading. Symptoms occurring from other bacterial infections like *Treponema pallidum* or *Haemphilus ducreyi* could be confused with HSV resulting in wrong diagnosis 115. Genital herpes may also cause atypical symptoms that occur at unusual sites such as the thighs or the buttocks. HSV-2 is also found to be a co-factor for HIV-1, which is one of the leading causes of sexually transmitted infections and at times it becomes difficult to diagnose the symptoms that occur due to HIV-1 co-infections 116. Hence, along with clinical diagnosis, laboratory tests are required to accurately diagnose genital herpes. To determine the presence of HSV in laboratory, swabs from the genital lesions are taken and tested by the following common techniques:

**i.** Viral culture of HSV has been a gold standard for laboratory diagnosis of HSV for the past two decades. Using the swabs from the genital lesions, the virus can be grown on tissue culture, usually within 5 days, that is then detected using immunofluorescence assays or by enzyme immunoassay. The limitation with this method is that it lacks sensitivity as more viruses are usually obtained from patients with primary infection (80%) but less from patients with recurrent infections (20-50%) or patients whose lesions have begun to heal 117.

**ii.** **Polymerase Chain Reaction (PCR):** This method of nucleic acid amplification has emerged as the next common method to assess the presence of HSV. Determining HSV by PCR is faster and four times more sensitive compared to viral culture 118,119. Based on this method, three assays have been approved by the US Food and Drug Association for the detection of HSV in genital lesions. These include IsoAmp HSV Assay, BioHelix Corporation; MultiCode-RTx Herpes Simplex Virus 1 & 2 Kit, EraGen Biosciences, Inc. and BD ProbeTec Herpes Simplex Viruses (HSV I & 2) QX Amplified DNA Assays, BD Diagnostic Systems. With increasing technology and advances in kit developments for HSV detection and typing using PCR, this method is rapidly replacing the viral culture assay.

**iii.** **Serotyping:** This method can not only be used to detect the presence of HSV but can also be used to differentiate between genital herpes originating from HSV-1 or HSV-2. Type-specific IgG against the glycoprotein G (gG) of HSV-1 and HSV-2 are available that can be used to distinguish between the two viruses 120. Serotyping has another advantage in that it detects the presence of HSV to confirm if the infection is a primary or recurrent infection. In primary infection, type-specific HSV antibodies can take from 2 weeks to 3 months to develop. Therefore, an initial absence of IgG antibodies specific for gG and subsequent development of such antibodies after 12 weeks confirms new HSV infection. Clinicians also recommend this method to diagnose genital herpes when there are no lesions or the above mentioned detection tests do not provide substantial results.

While this review only mentions the above common techniques to diagnose genital herpes in a laboratory setting, there are currently other methods and techniques being developed by research institutes and companies. For example, LeGoff *et al*. provide a detailed description of other available and upcoming diagnostic methods 121.

## TREATMENT AND PREVENTION

Genital herpes conditions are primarily treated with antivirals that aim at controlling viral replication. Acyclovir, its analogue Valacyclovir and Famcyclovir (prodrug of Pencyclovir) are currently prescribed for genital herpes treatment. These drugs are nucleoside analogues that specifically inhibit the herpesvirus DNA polymerase. While cyclovir is available in oral and intravenous formulations, Valacyclovir and Famcyclovir are available only as oral formulations. For primary infections where the symptoms can be severe, antiviral therapy is usually started even before the symptoms are confirmed by laboratory diagnosis and the duration of the therapy is 7-10 days or till the lesions are healed 122. In severe cases, to relieve pain, clinicians recommend the use of analgesics or sitz baths where the patients’ hips and buttocks are immersed in lukewarm water 117.

Preventive strategies to efficiently reduce the transmission of the virus also exist and in combination with the above mentioned treatments there could probably be a significant reduction of viral transmission. In the case of people that have symptomatic viral shedding, the most common preventive strategy is to abstain from sexual activity or to use condoms. A prospective study showed significantly lowered levels of viral acquisition among partners that used male condoms 123. Although it is thought that female condoms can also reduce virus transmission, this has not been clinically investigated. Applications of topical microbicides to prevent genital herpes infections are also being investigated. This strategy involves the use of natural or synthetic products that either increase the natural vaginal defenses or inactivate the HSV virions 124,125. A recent study showed that vaginal application of tenofovir gel, an antiviral microbicide which functions as a nucleotide reverse-transcriptase inhibitor, reduced the levels of HSV-2 acquisition among women in South Africa 5.

Various other therapeutic and prevention strategies that target different stages of virus lifecycle are currently being investigated. Peptide therapeutics is fast rising owing to the ease of synthesis, modifications and their high specificity 126. They are being synthesized and used as inhibitors against HSV infections 127. The TAT (transactivator of transcription)-peptide, derived from HIV, has been shown to inhibit infection of HSV in the *in vitro* and *in vivo* models of HSV infections 128,129. A study showed the effect of a synthetic 3-OS HS specific peptide: G2 in blocking HSV-2 infections in human cervical (HeLa) cell lines. This peptide significantly blocked the entry and thereby the spread of the virus 130 and a D-enantiomer of this peptide exhibits higher stability and more promise in inhibiting HSV infection 131. Another study designed synthetic peptides specific to the glycoproteins gD and gG and showed that these peptides can effectively recognize HSV-2 antibodies and hence may be used for serodiagnostic assays 132. Because HSV utilizes the cytoskeleton filaments and kinases during its entry, a recent study showed that blocking the myosin light chain kinase (MLCK) with inhibitors such as blebbistatin significantly reduces HSV infection 133, providing new evidence for potential targets in blocking HSV infections. The advent of nanoparticles in drug delivery was successful, owing to their ability to provide sustained or extended delivery of drugs at a local site. Nanoparticles or nanoparticle compositions to protect against HSV-2 infections are also being actively researched. Zinc Oxide (ZnO) nanoparticles exhibited significant antiviral activity in both the *in vitro* model using vaginal epithelial cells and the *in vivo* mice model of HSV-2 infections 134. Three different modes of treatment were used in this study: prophylaxis, therapeutic and neutralization. In all the three modes of treatment, the ZnO nanoparticles showed promising results in blocking HSV-2 infections. Another study showed the potential antiviral use of mucus-penetrating nanoparticles 135. In this study, acyclovir monophosphate loaded mucus-penetrating nanoparticles showed an increase in drug retention and distribution thereby providing an effective protection against HSV-2 challenge.

Protection against genital herpes infections can be enhanced by induction of protective immune responses using vaccines. Vaccines against genital herpes are underway and in the majority of clinical trials only prophylactic vaccines have seen success so far. There have been no reports of any therapeutic vaccines that show promise against genital herpes infections. These vaccines consist of subunits of glycoproteins such as gD or gB. A gD2 subunit vaccine, when administered with alum as adjuvant, showed around 39-46% efficacy in preventing HSV-2 infections in patients that were seronegative for HSV-1 and HSV-2 but did not provide protection to patients that were seropositive for HSV-1 136,137. Other viral glycoproteins such as gC and gE are also being used as vaccines to study their effectivity in blocking genital herpes infections 138,139. Peptide based vaccines are also being developed to incite immune responses against HSV-2 infections. A study developed a peptide based vaccine: HerpV, which generates CD4^+^ and CD8^+^ responses when subjected to HSV-2 challenge 140,141.

## CONCLUSION AND FUTURE DIRECTIONS

There is no doubt that our understanding of HSV-2 lifecycle and associated pathogenesis has improved dramatically over the last several years but challenges remain in many areas, especially those relating to disease management and prevention. The new knowledge has provided a major opportunity to develop new strategies for patient care by combining our understanding of viral infection mechanisms, host immune responses, and the viral mechanisms that subvert them. New anti-HSV drugs are on the horizon, many of which may target other herpesviruses as well. At present, the development of vaccines against HSV-2 is a highly active area of research and many innovative strategies are currently being tested for an effective vaccine generation. Future clinical trials will see many new, non-nucleoside anti-herpetic drug candidates as well as many newer approaches, including immune-based therapeutics. An area that needs extra attention is rapid diagnostics, especially since genital herpes can be caused by both HSV-1 and HSV-2. Therefore quick and easily available tests can yield much better results in reducing symptoms and lowering transmission rate. Any success in reducing transmission rate will mean a step closer to the greatest challenge for herpes virologists, which is complete elimination of this lifelong infection.

## References

[1] Wald A (2006). Genital HSV-1 infections.. Sex Transm Infect.

[2] Felman YM, Nikitas JA (1983). Sexually transmitted diseases and child sexual abuse. Part II.. N Y State J Med.

[3] Kinghorn GR (1993). Genital herpes: natural history and treatment of acute
episodes.. J Med Virol Suppl.

[4] Abdool Karim SS, Abdool Karim Q, Kharsany ABM, Baxter C, Grobler AC, Werner L, Kashuba A, Mansoor LE, Samsunder N, Mindel A, Gengiah TN (2015). Tenofovir Gel for the Prevention of Herpes Simplex Virus Type 2
Infection.. N Engl J Med.

[5] Wald A, Corey L (2007). Persistence in the population: epidemiology,
transmission.. In: Arvin A, Campadelli-Fiume G, Mocarski E, Moore PS, Roizman B,
Whitley R, Yamanishi K, editors. Human Herpesviruses: Biology, Therapy, and
Immunoprophylaxis..

[6] Xu F, Schillinger JA, Sternberg MR, Johnson RE, Lee FK, Nahmias AJ, Markowitz LE (2002). Seroprevalence and Coinfection with Herpes Simplex Virus Type 1
and Type 2 in the United States, 1988-1994.. J Infect Dis.

[7] Huengsberg M (2000). Sexually Transmitted Diseases.. Sex Transm Infect.

[8] Halpern-Felsher BL, Cornell JL, Kropp RY, Tschann JM (2005). Oral versus vaginal sex among adolescents: per-ceptions,
attitudes, and behavior.. Pediatrics.

[9] Roberts CM, Pfister JR, Spear SJ (2003). Increasing proportion of herpes simplex virus type 1 as a cause
of genital herpes infection in college students.. Sex Transm Dis.

[10] Scoular A, Norrie J, Gillespie G, Mir N, Carman WF (2002). Longitudinal study of genital infection by herpes simplex virus
type 1 in western Scotland over 15 years.. BMJ.

[11] Manavi K, McMillan A, Ogilvie M (2004). Herpes simplex virus type 1 remains the principal cause of
initial anogenital herpes in Edinburgh, Scotland.. Sex Transm Dis.

[12] Buxbaum S, Geers M, Gross G, Schofer H, Rabenau HF, Doerr HW (2003). Epidemiology of herpes simplex virus types 1 and 2 in Germany:
what has changed?. Med Microbiol Immunol (Berl).

[13] U.S. Departement of Health, Education, and Welfare, Publich Health
Service, Center for Disease Control (2010). Morbidity and Mortality Weekly Report: MMWR
(2010).. http://www.cdc.gov/mmwr/preview/mmwrhtml/mm5915a3.htm.

[14] Malkin J-E (2004). Epidemiology of genital herpes simplex virus infection in
developed countries.. Herpes J IHMF.

[15] Xu F, Sternberg MR, Kottiri BJ, McQuillan GM, Lee FK, Nahmias AJ, Berman SM, Markowitz LE (2006). Trends in herpes simplex virus type 1 and type 2 seroprevalence
in the United States.. JAMA.

[16] Weiss HA, Buve A, Robinson NJ, Van Dyck E, Kahindo M, Anagonou S, Musonda R, Zekeng L, Morison L, Carael M, Laga M, Hayes RJ (2001). The epidemiology of HSV-2 infection and its association with HIV
infection in four urban African populations.. AIDS Lond Engl 15 Suppl.

[17] Weiss H (2004). Epidemiology of herpes simplex virus type 2 infection in the
developing world.. Herpes J IHMF.

[18] Watson-Jones D, Weiss HA, Rusizoka M, Baisley K, Mugeye K, Changalucha J, Everett D, Balira R, Knight L, Ross D, Hayes RJ (2007). Risk factors for herpes simplex virus type 2 and HIV among women
at high risk in northwest-ern Tanzania: preparing for an HSV-2 intervention
trial.. J Acquir Immune Defic Syndr 1999.

[19] Shukla D, Spear PG (2001). Herpesviruses and heparan sulfate: an intimate relationship in
aid of viral entry.. J Clin Invest.

[20] Herold BC, WuDunn D, Soltys N, Spear PG (1991). Glycoprotein C of herpes simplex virus type 1 plays a prin-cipal
role in the adsorption of virus to cells and in infectivity.. J Virol.

[21] Spear PG, Eisenberg RJ, Cohen GH (2000). Three classes of cell surface receptors for alphaherpesvirus
entry.. Virology.

[22] Dingwell KS, Johnson DC (1998). The Herpes Simplex Virus gE-gI Complex Facilitates Cell-to-Cell
Spread and Binds to Components of Cell Junctions.. J Virol.

[23] Weissenhorn W, Hinz A, Gaudin Y (2007). Virus membrane fusion.. Membr Traffick.

[24] Friedman HM, Cohen GH, Eisenberg RJ, Seidel CA, Cines DB (1984). Glycoprotein C of herpes simplex virus 1 acts as a receptor for
the C3b complement component on infected cells.. Nature.

[25] Baucke RB, Spear PG (1979). Membrane proteins specified by herpes simplex viruses. V.
Identification of an Fc-binding glycoprotein.. J Virol.

[26] Johnson DC, Feenstra V (1987). Identification of a novel herpes simplex virus type 1-induced
glycoprotein which complexes with gE and binds
immunoglobulin.. J Virol.

[27] Johnson DC, Frame MC, Ligas MW, Cross AM, Stow ND (1988). Herpes simplex virus immunoglobulin G Fc receptor activity
depends on a complex of two viral glycoproteins, gE and gI.. J Virol.

[28] David AT, Baghian A, Foster TP, Chouljenko VN, Kousoulas KG (2008). The herpes simplex virus type 1 (HSV-1) glycoprotein K(gK) is
essential for viral corneal spread and neuroinvasiveness.. Curr Eye Res.

[29] Hutchinson L, Johnson DC (1995). Herpes simplex virus glycoprotein K promotes egress of virus
particles.. J Virol.

[30] Kim I-J, Chouljenko VN, Walker JD, Kousoulas KG (2013). Herpes simplex virus 1 glycoprotein M and the membrane-associated
protein UL11 are required for virus-induced cell fusion and efficient virus
entry.. J Virol.

[31] Baines JD, Wills E, Jacob RJ, Pennington J, Roizman B (2007). Glycoprotein M of Herpes Simplex Virus 1 Is Incorporated into
Virions during Budding at the Inner Nuclear Membrane.. J Virol.

[32] Lau KS-Y, Crump MC (2015). HSV-1 gM and the gK/pUL20 Complex Are Important for the
Localization of gD and gH/L to Viral Assembly Sites.. Viruses.

[33] Herold BC, Visalli RJ, Susmarski N, Brandt CR, Spear PG (1994). Glycoprotein C-independent binding of herpes simplex virus to
cells requires cell surface heparan sulphate and glycoprotein
B.. J Gen Virol.

[34] Oh M-J, Akhtar J, Desai P, Shukla D (2010). A role for heparan sulfate in viral surfing.. Biochem Biophys Res Commun.

[35] Spear M, Wu Y (2014). Viral exploitation of actin: force-generation and scaffolding
functions in viral infection.. Virol Sin.

[36] Spear PG, Eisenberg RJ, Cohen GH (2000). Three classes of cell surface receptors for alphaherpesvirus
entry.. Virology.

[37] Liu J, Pedersen LC (2007). Anticoagulant heparan sulfate: structural specificity and
biosynthesis.. Appl Microbiol Biotechnol.

[38] Shukla D, Liu J, Blaiklock P, Shworak NW, Bai X, Esko JD, Cohen GH, Eisenberg RJ, Rosenberg RD, Spear PG (1999). A novel role for 3-O-sulfated heparan sulfate in herpes simplex
virus 1 entry.. Cell.

[39] Tiwari V, O’donnell C, Copeland RJ, Scarlett T, Liu J, Shukla D (2007). Soluble 3-O-sulfated heparan sulfate can trigger herpes simplex
virus type 1 entry into resistant Chinese hamster ovary (CHO-K1)
cells.. J Gen Virol.

[40] Nicola AV, Hou J, Major EO, Straus SE (2005). Herpes simplex virus type 1 enters human epidermal
keratino-cytes, but not neurons, via a pH-dependent endocytic
pathway.. J Virol.

[41] Nicola AV, McEvoy AM, Straus SE (2003). Roles for endocytosis and low pH in herpes simplex virus entry
into HeLa and Chinese hamster ovary cells.. J Virol.

[42] Cai WZ, Person S, Warner SC, Zhou JH, DeLuca NA (1987). Linker-insertion nonsense and restriction-site dele-tion
mutations of the gB glycoprotein gene of herpes simplex virus type
1.. J Virol.

[43] Satoh T, Arii J, Suenaga T, Wang J, Kogure A, Uehori J, Arase N, Shiratori I, Tanaka S, Kawaguchi Y, Spear PG, Lanier LL, Arase H (2008). PILRalpha is a herpes simplex virus-1 entry coreceptor that
associates with glycoprotein B.. Cell.

[44] Arii J, Wang J, Morimoto T, Suenaga T, Akashi H, Arase H, Kawaguchi Y (2010). A single-amino-acid substitu-tion in herpes simplex virus 1
envelope glycoprotein B at a site required for binding to the paired
immunoglobulin-like type 2 receptor alpha (PILRalpha) abrogates
PILRalpha-dependent viral entry and reduces pathogenesis.. J Virol.

[45] Wang J, Fan Q, Satoh T, Arii J, Lanier LL, Spear PG, Kawaguchi Y, Arase H (2009). Binding of herpes simplex virus glycoprotein B (gB) to paired
immunoglobulin-like type 2 receptor alpha depends on specific sialylated
O-linked glycans on gB.. J Virol.

[46] Suenaga T, Satoh T, Somboonthum P, Kawaguchi Y, Mori Y, Arase H (2010). Myelin-associated glycoprotein mediates membrane fusion and entry
of neurotropic herpesviruses.. Proc Natl Acad Sci U S A.

[47] Arii J, Goto H, Suenaga T, Oyama M, Kozuka-Hata H, Imai T, Minowa A, Akashi H, Arase H, Kawaoka Y, Kawaguchi Y (2010). Non-muscle myosin IIA is a functional entry receptor for herpes
simplex virus-1.. Nature.

[48] Handler CG, Eisenberg RJ, Cohen GH (1996). Oligomeric structure of glycoproteins in herpes simplex virus
type 1.. J Virol.

[49] Handler CG, Cohen GH, Eisenberg RJ (1996). Cross-linking of glycoprotein oligomers during herpes simplex
virus type 1 entry.. J Virol.

[50] Roop C, Hutchinson L, Johnson DC (1993). A mutant herpes simplex virus type 1 unable to express
glycopro-tein L cannot enter cells, and its particles lack glycoprotein
H.. J Virol.

[51] Hutchinson L, Browne H, Wargent V, Davis-Poynter N, Primorac S, Goldsmith K, Minson AC, Johnson DC (1992). A novel herpes simplex virus glycoprotein, gL, forms a complex
with glycoprotein H (gH) and affects normal folding and surface expression
of gH.. J Virol.

[52] Browne H, Baxter V, Minson T (1993). Analysis of protective immune responses to the glycoprotein
H-glycoprotein L complex of herpes simplex virus type 1.. J Gen Virol.

[53] Gianni T, Cerretani A, Dubois R, Salvioli S, Blystone SS, Rey F, Campadelli-Fiume G (2010). Herpes simplex virus glycoproteins H/L bind to cells
independently of {alpha}V{beta}3 integrin and inhibit virus entry, and their
consti-tutive expression restricts infection.. J Virol.

[54] Cheshenko N, Trepanier JB, Gonzalez PA, Eugenin EA, Jacobs WRJ, Herold BC (2014). Herpes simplex virus type 2 glycoprotein H interacts with
integrin alphavbeta3 to facilitate viral entry and calcium signaling in
human genital tract epithelial cells.. J Virol.

[55] Gianni T, Salvioli S, Chesnokova LS, Hutt-Fletcher LM, Campadelli-Fiume G (2013). αvβ6- and αvβ8-Integrins Serve As Interchangeable Receptors for
HSV gH/gL to Promote Endocytosis and Activation of Membrane
Fusion.. PLoS Pathog.

[56] Gianni T, Massaro R, Campadelli-Fiume G (2015). Dissociation of HSV gL from gH by alphavbeta6- or
al-phavbeta8-integrin promotes gH activation and virus
entry.. Proc Natl Acad Sci U S A.

[57] Clement C, Tiwari V, Scanlan PM, Valyi-Nagy T, Yue BYJT, Shukla D (2006). A novel role for phagocytosis-like uptake in herpes simplex virus
entry.. J Cell Biol.

[58] Akhtar J, Shukla D (2009). Viral entry mechanisms: cellular and viral mediators of herpes
simplex virus entry.. FEBS J.

[59] Tiwari V, Shukla D (2010). Phosphoinositide 3 kinase signalling may affect multiple steps
during herpes simplex virus type-1 entry.. J Gen Virol.

[60] Zheng K, Xiang Y, Wang X, Wang Q, Zhong M, Wang S, Wang X, Fan J, Kitazato K, Wang Y (2014). Epidermal Growth Factor Receptor-PI3K Signaling Controls Cofilin
Activity To Facilitate Herpes Simplex Virus 1 Entry into Neuronal Cells.. mBio.

[61] Cheshenko N, Trepanier JB, Stefanidou M, Buckley N, Gonzalez P, Jacobs W, Herold BC (2013). HSV activates Akt to trigger calcium release and promote viral
entry: novel candidate target for treatment and suppression.. FASEB J Off Publ Fed Am Soc Exp Biol.

[62] Dohner K, Wolfstein A, Prank U, Echeverri C, Dujardin D, Vallee R, Sodeik B (2002). Function of dynein and dynactin in herpes simplex virus capsid
transport.. Mol Biol Cell.

[63] Radtke K, Kieneke D, Wolfstein A, Michael K, Steffen W, Scholz T, Karger A, Sodeik B (2010). Plus- and Minus-End Directed Microtubule Motors Bind
Simultaneously to Herpes Simplex Virus Capsids Using Different Inner
Tegument Structures.. PLoS Pathog.

[64] Sodeik B, Ebersold MW, Helenius A (1997). Microtubule-mediated transport of incoming herpes simplex virus 1
capsids to the nucleus.. J Cell Biol.

[65] Zhong M, Zheng K, Chen M, Xiang Y, Jin F, Ma K, Qiu X, Wang Q, Peng T, Kitazato K, Wang Y (2014). Heat-Shock Protein 90 Promotes Nuclear Transport of Herpes
Simplex Virus 1 Capsid Protein by Interacting with Acetylated
Tubulin.. PLoS ONE.

[66] He B, Gross M, Roizman B (1997). The gamma(1)34. protein of herpes simplex virus 1 complexes with
protein phosphatase 1alpha to dephosphorylate the alpha subunit of the
eukaryotic translation initiation factor 2 and preclude the shutoff of
protein synthesis by double-stranded RNA-activated protein
kinase.. Proc Natl Acad Sci U S A.

[67] Talloczy Z, Virgin HW 4th, Levine B (2006). PKR-dependent autophagic degradation of herpes simplex virus type
1.. Autophagy.

[68] Orvedahl A, Alexander D, Talloczy Z, Sun Q, Wei Y, Zhang W, Burns D, Leib DA, Levine B (2007). HSV-1 ICP34.5 confers neurovirulence by targeting the Beclin 1
autophagy protein.. Cell Host Microbe.

[69] Yakoub AM, Shukla D (2015). Basal Autophagy Is Required for Herpes simplex Virus-2
Infection.. Sci Rep.

[70] Choi E-J, Kee S-H (2014). Axin expression delays herpes simplex virus-induced autophagy and
enhances viral replication in L929 cells.. Microbiol Immunol.

[71] Roizman B, Whitley RJ (2013). An inquiry into the molecular basis of HSV latency and
reactivation.. Annu Rev Microbiol.

[72] Kramer MF, Chen SH, Knipe DM, Coen DM (1998). Accumulation of viral transcripts and DNA during estab-lishment
of latency by herpes simplex virus.. J Virol.

[73] Kramer MF, Coen DM (1995). Quantification of transcripts from the ICP4 and thymidine kinase
genes in mouse ganglia latently infected with herpes simplex
virus.. J Virol.

[74] Kent JR, Kang W, Miller CG, Fraser NW (2003). Herpes simplex virus latency-associated transcript gene
function.. J Neurovirol.

[75] Perng GC, Jones C, Ciacci-Zanella J, Stone M, Henderson G, Yukht A, Slanina SM, Hofman FM, Ghiasi H, Nesburn AB, Wechsler SL (2000). Virus-induced neuronal apoptosis blocked by the herpes simplex
virus latency-associated transcript.. Science.

[76] Shimeld C, Whiteland JL, Nicholls SM, Grinfeld E, Easty DL, Gao H, Hill TJ (1995). Immune cell infiltration and persistence in the mouse trigeminal
ganglion after infection of the cornea with herpes simplex virus type
1.. J Neuro-immunol.

[77] Halford WP, Gebhardt BM, Carr DJ (1996). Persistent cytokine expression in trigeminal ganglion latently
infect-ed with herpes simplex virus type 1.. J Immunol.

[78] Liu T, Tang Q, Hendricks RL (1996). Inflammatory infiltration of the trigeminal ganglion after herpes
simplex virus type 1 corneal infection.. J Virol.

[79] Chen SH, Garber DA, Schaffer PA, Knipe DM, Coen DM (2000). Persistent elevated expression of cytokine transcripts in ganglia
latently infected with herpes simplex virus in the absence of ganglionic
replication or reactivation.. Virology.

[80] Liu T, Khanna KM, Chen X, Fink DJ, Hendricks RL (2000). CD8(+) T cells can block herpes simplex virus type 1 (HSV-1)
reactivation from latency in sensory neurons.. J Exp Med.

[81] Wilcox CL, Johnson EM (1987). Nerve growth factor deprivation results in the reactivation of
latent herpes sim-plex virus in vitro.. J Virol.

[82] Hill JM, Garza HHJ, Helmy MF, Cook SD, Osborne PA, Johnson EMJ, Thompson HW, Green LC, O’Callaghan RJ, Gebhardt BM (1997). Nerve growth factor antibody stimulates reactivation of ocular
herpes simplex virus type 1 in latently infected rabbits.. J Neurovirol.

[83] Kristie TM, Vogel JL, Sears AE (1999). Nuclear localization of the C1 factor (host cell factor) in
sensory neurons correlates with reactivation of herpes simplex virus from
latency.. Proc Natl Acad Sci U S A.

[84] Dressler GR, Rock DL, Fraser NW (1987). Latent herpes simplex virus type 1 DNA is not extensively
methylated in vivo.. J Gen Virol.

[85] Kubat NJ, Tran RK, McAnany P, Bloom DC (2004). Specific histone tail modification and not DNA methylation is a
determinant of herpes simplex virus type 1 latent gene
expression.. J Virol.

[86] Oh J, Fraser NW (2008). Temporal Association of the Herpes Simplex Virus Genome with
Histone Proteins during a Lytic Infection.. J Virol.

[87] Deshmane SL, Fraser NW (1989). During latency, herpes simplex virus type 1 DNA is associated
with nucleo-somes in a chromatin structure.. J Virol.

[88] Kent JR, Zeng P-Y, Atanasiu D, Gardner J, Fraser NW, Berger SL (2004). During lytic infection herpes simplex virus type 1 is associated
with histones bearing modifications that correlate with active
transcription.. J Virol.

[89] Knipe DM, Cliffe A (2008). Chromatin control of herpes simplex virus lytic and latent
infection.. Nat Rev Micro.

[90] Kubat NJ, Amelio AL, Giordani NV, Bloom DC (2004). The herpes simplex virus type 1 latency-associated tran-script
(LAT) enhancer/rcr is hyperacetylated during latency independently of LAT
transcription.. J Virol.

[91] Wang Q-Y, Zhou C, Johnson KE, Colgrove RC, Coen DM, Knipe DM (2005). Herpesviral latency-associated transcript gene promotes assembly
of heterochromatin on viral lytic-gene promoters in latent
infection.. Proc Natl Acad Sci U S A.

[92] Amelio AL, McAnany PK, Bloom DC (2006). A chromatin insulator-like element in the herpes simplex virus
type 1 latency-associated transcript region binds CCCTC-binding factor and
displays enhancer-blocking and silencing activities.. J Virol.

[93] Sun L, Li Q (2012). The miRNAs of herpes simplex virus (HSV).. Virol Sin.

[94] Reynolds AE, Ryckman BJ, Baines JD, Zhou Y, Liang L, Roller RJ (2001). U(L)31 and U(L)34 Proteins of Her-pes Simplex Virus Type 1 Form a
Complex That Accumulates at the Nuclear Rim and Is Required for Envelopment
of Nucleocapsids.. J Virol.

[95] Collins WJ, Johnson DC (2003). Herpes simplex virus gE/gI expressed in epithelial cells
interferes with cell-to-cell spread.. J Virol.

[96] Hadigal SR, Agelidis AM, Karasneh GA, Antoine TE, Yakoub AM, Ramani VC, Djalilian AR, Sanderson RD, Shukla D (2015). Heparanase is a host enzyme required for herpes simplex virus-1
release from cells.. Nat Commun.

[97] van Leeuwen H, Elliott G, O’Hare P (2002). Evidence of a role for nonmuscle myosin II in herpes simplex
virus type 1 egress.. J Virol.

[98] Roberts KL, Baines JD (2010). Myosin Va enhances secretion of herpes simplex virus 1 virions
and cell surface expression of viral glycoproteins.. J Virol.

[99] Lafferty WE, Downey L, Celum C, Wald A (2000). Herpes simplex virus type 1 as a cause of genital herpes: impact
on surveillance and prevention.. J Infect Dis.

[100] Mertz GJ, Rosenthal SL, Stanberry LR (2003). Is herpes simplex virus type 1 (HSV-1) now more common than HSV-2
in first episodes of genital herpes?. Sex Transm Dis.

[101] Lowhagen GB, Tunback P, Andersson K, Bergstrom T, Johannisson G (2000). First episodes of genital herpes in a Swedish STD population: a
study of epidemiology and transmission by the use of herpes simplex virus
(HSV) typing and specific serology.. Sex Transm Infect.

[102] Bernstein DI, Lovett MA, Bryson YJ (1984). Serologic analysis of first-episode nonprimary genital herpes
sim-plex virus infection. Presence of type 2 antibody in acute serum
samples.. Am J Med.

[103] Corey L, Spear PG (1986). Infections with herpes simplex viruses (1).. N Engl J Med.

[104] Corey L, Adams HG, Brown ZA, Holmes KK (1983). Genital herpes simplex virus infections: clinical
manifes-tations, course, and complications.. Ann Intern Med.

[105] Zhu J, Koelle DM, Cao J, Vazquez J, Huang ML, Hladik F, Wald A, Corey L (2007). Virus-specific CD8+ T cells accumulate near sensory nerve endings
in genital skin during subclinical HSV-2 reactivation.. J Exp Med.

[106] Zhu J, Hladik F, Woodward A, Klock A, Peng T, Johnston C, Remington M, Magaret A, Koelle DM, Wald A, Corey L (2009). Persistence of HIV-1 receptor-positive cells after HSV-2
reactivation is a potential mechanism for in-creased HIV-1
acquisition.. Nat Med.

[107] Johnson KE, Redd AD, Quinn TC, Collinson-Streng AN, Cornish T, Kong X, Sharma R, Tobian AAR, Tsai B, Sherman ME, Kigozi G, Serwadda D, Wawer MJ, Gray RH (2011). Effects of HIV-1 and herpes simplex virus type 2 infection on
lymphocyte and dendritic cell density in adult foreskins from Rakai,
Uganda.. J Infect Dis.

[108] Cunningham AL, Diefenbach RJ, Miranda-Saksena M, Bosnjak L, Kim M, Jones C, Douglas MW (2006). The cycle of human herpes simplex virus infection: virus
transport and immune control.. J Infect Dis.

[109] Liu T, Khanna KM, Carriere BN, Hendricks RL (2001). Gamma interferon can prevent herpes simplex virus type 1
reactivation from latency in sensory neurons.. J Virol.

[110] Donaghy H, Bosnjak L, Harman AN, Marsden V, Tyring SK, Meng T-C, Cunningham AL (2009). Role for plasmacytoid dendritic cells in the immune control of
recurrent human herpes simplex virus infection.. J Virol.

[111] Mott KR, Allen SJ, Zandian M, Konda B, Sharifi BG, Jones C, Wechsler SL, Town T, Ghiasi H (2014). CD8α Dendritic Cells Drive Establishment of HSV-1
Latency.. PLoS ONE.

[112] Mott KR, Allen SJ, Zandian M, Ghiasi H (2014). Coregulatory interactions among CD8alpha dendritic cells, the
latency-associated transcript, and programmed death 1 contribute to higher
levels of herpes simplex virus 1 latency.. J Virol.

[113] Koelle DM, Chen HB, Gavin MA, Wald A, Kwok WW, Corey L (2001). CD8 CTL from genital herpes simplex lesions: recognition of viral
tegument and immediate early proteins and lysis of infected cutaneous
cells.. J Immunol Bal-tim Md 1950.

[114] Koelle DM, Frank JM, Johnson ML, Kwok WW (1998). Recognition of herpes simplex virus type 2 tegument proteins by
CD4 T cells infiltrating human genital herpes lesions.. J Virol.

[115] Mackay IM, Harnett G, Jeoffreys N, Bastian I, Sriprakash KS, Siebert D, Sloots TP (2006). Detection and dis-crimination of herpes simplex viruses,
Haemophilus ducreyi, Treponema pallidum, and Calymmatobacterium
(Klebsiel-la) granulomatis from genital ulcers.. Clin Infect Dis Off Publ Infect Dis Soc Am.

[116] Corey L, Wald A, Celum CL, Quinn TC (2004). The effects of herpes simplex virus-2 on HIV-1 acquisition and
transmission: a review of two overlapping epidemics.. J Acquir Immune Defic Syndr 1999.

[117] Gupta R, Warren T, Wald A (2007). Genital herpes.. Lancet Lond Engl.

[118] Ramaswamy M, McDonald C, Smith M, Thomas D, Maxwell S, Tenant-Flowers M, Geretti AM (2004). Diag-nosis of genital herpes by real time PCR in routine clinical
practice.. Sex Transm Infect.

[119] Filen F, Strand A, Allard A, Blomberg J, Herrmann B (2004). Duplex real-time polymerase chain reaction assay for detection
and quantification of herpes simplex virus type 1 and herpes simplex virus
type 2 in genital and cutaneous lesions.. Sex Transm Dis.

[120] Lafferty WE, Krofft S, Remington M, Giddings R, Winter C, Cent A, Corey L (1987). Diagnosis of herpes sim-plex virus by direct immunofluorescence
and viral isolation from samples of external genital lesions in a
high-prevalence population.. J Clin Microbiol.

[121] LeGoff J, Pere H, Belec L (2014). Diagnosis of genital herpes simplex virus infection in the
clinical laboratory.. Virol J.

[122] Workowski K (2015). Sexually Transmitted Diseases Treatment Guidelines, 2015.. http://www.cdc.gov/mmwr/preview/mmwrhtml/rr6403a1.htm.

[123] Wald A, Langenberg AGM, Krantz E, Douglas JMJ, Handsfield HH, DiCarlo RP, Adimora AA, Izu AE, Morrow RA, Corey L (2005). The relationship between condom use and herpes simplex virus
acquisition.. Ann Intern Med.

[124] Keller MJ, Tuyama A, Carlucci MJ, Herold BC (2005). Topical microbicides for the prevention of genital her-pes
infection.. J Antimicrob Chemother.

[125] Yang D, Chertov O, Oppenheim JJ (2001). Participation of mammalian defensins and cathelicidins in
anti-microbial immunity: receptors and activities of human defensins and
cathelicidin (LL-37).. J Leukoc Biol.

[126] Lien S, Lowman HB (2003). Therapeutic peptides.. Trends Biotechnol.

[127] Galdiero S, Falanga A, Tarallo R, Russo L, Galdiero E, Cantisani M, Morelli G, Galdiero M (2013). Peptide inhibitors against herpes simplex virus
infections.. J Pept Sci Off Publ Eur Pept Soc.

[128] Jose GG, Larsen IV, Gauger J, Carballo E, Stern R, Brummel R, Brandt CR (2013). A Cationic Peptide, TAT-Cd(0), Inhibits Herpes Simplex Virus Type
1 Ocular Infection In Vivo.. Invest Ophthalmol Vis Sci.

[129] Larsen IV, Brandt CR (2010). A Cationic TAT Peptide Inhibits Herpes Simplex Virus Type 1
Infection of Hu-man Corneal Epithelial Cells.. J Ocul Pharmacol Ther.

[130] Ali MM, Karasneh GA, Jarding MJ, Tiwari V, Shukla D (2012). A 3-O-sulfated heparan sulfate binding peptide preferentially
targets herpes simplex virus 2-infected cells.. J Virol.

[131] Jaishankar D, Yakoub AM, Bogdanov A, Valyi-Nagy T, Shukla D (2015). Characterization of a proteolytically stable D-peptide that
suppresses herpes simplex virus 1 infection: implications for the
development of entry-based antiviral therapy.. J Virol.

[132] Levi M, Ruden U, Carlberg H, Wahren B (1999). The use of peptides from glycoproteins G-2 and D-1 for de-tecting
herpes simplex virus type 2 and type-common antibodies.. J Clin Virol Off Publ Pan Am Soc Clin Virol.

[133] Antoine TE, Shukla D (2014). Inhibition of myosin light chain kinase can be targeted for the
development of new therapies against herpes simplex virus type-1
infection.. Antivir Ther.

[134] Antoine TE, Mishra YK, Trigilio J, Tiwari V, Adelung R, Shukla D (2012). Prophylactic, therapeutic and neu-tralizing effects of zinc oxide
tetrapod structures against herpes simplex virus type-2
infection.. Antiviral Res.

[135] Ensign LM, Tang BC, Wang Y-Y, Tse TA, Hoen T, Cone R, Hanes J (2012). Mucus-penetrating nanoparticles for vaginal drug delivery protect
against herpes simplex virus.. Sci Transl Med.

[136] Straus SE, Wald A, Kost RG, McKenzie R, Langenberg AG, Hohman P, Lekstrom J, Cox E, Nakamura M, Sekulo-vich R, Izu A, Dekker C, Corey L (1997). Immunotherapy of recurrent genital herpes with recombinant herpes
sim-plex virus type 2 glycoproteins D and B: results of a placebo-controlled
vaccine trial.. J Infect Dis.

[137] Stanberry LR, Spruance SL, Cunningham AL, Bernstein DI, Mindel A, Sacks S, Tyring S, Aoki FY, Slaoui M, Denis M, Vandepapeliere P, Dubin G (2002). Glycoprotein-D-adjuvant vaccine to prevent genital
herpes.. N Engl J Med.

[138] Awasthi S, Mahairas GG, Shaw CE, Huang M-L, Koelle DM, Posavad C, Corey L, Friedman HM (2015). A Dual-Modality Herpes Simplex Virus 2 Vaccine for Preventing
Genital Herpes by Using Glycoprotein C and D Subunit Antigens To Induce
Potent Antibody Responses and Adenovirus Vectors Containing Capsid and
Tegument Proteins as T Cell Immunogens.. J Virol.

[139] Awasthi S, Huang J, Shaw C, Friedman HM (2014). Blocking herpes simplex virus 2 glycoprotein E immune evasion as
an approach to enhance efficacy of a trivalent subunit antigen vaccine for
genital herpes.. J Virol.

[140] Mo A, Musselli C, Chen H, Pappas J, Leclair K, Liu A, Chicz RM, Truneh A, Monks S, Levey DL, Srivastava PK (2011). A heat shock protein based polyvalent vaccine targeting HSV-2:
CD4(+) and CD8(+) cellular immunity and protective efficacy.. Vaccine.

[141] Wald A, Koelle DM, Fife K, Warren T, Leclair K, Chicz RM, Monks S, Levey DL, Musselli C, Srivastava PK (2011). Safety and immunogenicity of long HSV-2 peptides complexed with
rhHsc70 in HSV-2 seropositive persons.. Vaccine.

